# Accuracy of Urinary Neutrophil Gelatinase-Associated Lipocalin in Quantifying Acute Kidney Injury after Partial Nephrectomy in Patients with Normal Contralateral Kidney

**DOI:** 10.1371/journal.pone.0133675

**Published:** 2015-07-22

**Authors:** Kyo Chul Koo, Jung Hwa Hong, Hye Sun Lee, Seong Uk Jeh, Young Deuk Choi, Koon Ho Rha, Won Sik Ham

**Affiliations:** 1 Department of Urology, Urological Science Institute, Yonsei University College of Medicine, Seoul, Republic of Korea; 2 Biostatistics Collaboration Unit, Department of Research Affairs, Yonsei University College of Medicine, Seoul, Republic of Korea; School of Public Health of University of São Paulo, BRAZIL

## Abstract

**Background:**

To evaluate the efficacy of urinary neutrophil gelatinase-associated lipocalin (uNGAL) for predicting the degree of acute kidney injury (AKI) in patients following partial nephrectomy (PN).

**Methods:**

This prospective study included 176 patients who underwent open or laparoscopic PN for solid renal tumors between June 2013 and May 2014. Urine samples were collected preoperatively and at 3, 24, and 48 h after renal pedicle clamp removal. Changes in uNGAL levels were analyzed for all patients and between subgroups that were dichotomized based on preoperative eGFR values of <60 and ≥60 mL/min/1.73m^2^, open and laparoscopic surgery, and according to the onset of AKI. Linear mixed models were used to investigate preoperative and perioperative features associated with postoperative uNGAL and eGFR changes at 6 months postoperatively.

**Results:**

Among 146 patients included in the final analysis, 10 (6.8%) patients had preoperative eGFR <60 mL/min/1.73m^2^. In the overall group, uNGAL levels increased following PN. However, all subgroups demonstrated comparable changes in uNGAL levels over time. Multivariate analyses failed to reveal any correctable clinical features associated with postoperative uNGAL changes, whereas preoperative serum creatinine levels and the onset of AKI correlated with eGFR at 6 months postoperatively.

**Conclusions:**

uNGAL levels may increase following PN. However, it does not appear to be a useful marker for quantifying the degree of AKI or predicting postoperative renal function in patients with normal contralateral kidney and relatively good preoperative renal function. Further analysis is necessary to assess the usefulness of uNGAL in patients with poor preoperative renal function.

## Introduction

The American Urological Association Guidelines recommend partial nephrectomy (PN) as the treatment of choice for cT1 tumors, as PN has demonstrated to offer oncological control equal to that of radical nephrectomy (RN), and at the same time, preserve renal function [1,2]. Notwithstanding the advantages of PN with respect to renal function preservation, patients undergoing this procedure are prone to viable parenchymal loss and ischemic renal injury, with subsequent risks for acute kidney injury (AKI) and chronic kidney disease. Many studies have relied on serum creatinine (sCr) levels to objectively estimate the degree of AKI during PN and to predict postoperative long-term renal function [3–5]. However, relatively small changes in sCr levels compared to large and rapid changes in glomerular filtration rate (GFR) deter the accurate diagnosis of AKI and may underestimate the degree of injury in the early phases of AKI [6].

Previous studies have proposed neutrophil gelatinase-associated lipocalin (NGAL) as an attractive marker for the early identification of ischemic and/or tubular damage [7–10], and few representative studies have evaluated the usefulness of urinary NGAL (uNGAL) for quantifying AKI following PN [11–13]. Abassi et al. reported the usefulness of uNGAL as a marker for AKI following open PN and demonstrated its performance in quantifying the degree of AKI [11]. In contrast, other studies have reported negative results for uNGAL in assessing AKI in patients who underwent open PN [12,13]. However, these studies were limited by small numbers of patients and the inclusion of only open procedures, wherein renal injury is minimized by renal protective measures such as cold ischemia. Indeed, in the current era of minimally invasive surgery, a large proportion of PN procedures are performed via laparoscopic or robot-assisted approaches; in these settings, the risk of AKI increases owing to warm ischemia and increased intraoperative abdominal pressure resulting from pneumoperitoneum.

To further investigate the efficacy of uNGAL in quantifying AKI during and subsequent to PN, we assessed a relatively large cohort of patients for changes in uNGAL following open or laparoscopic PN and the clinical features associated with these changes. In order to assess renal functional changes in these patients, we also evaluated whether changes in uNGAL levels following PN or any clinical features were associated with estimated GFR (eGFR) at 6 months postoperatively.

## Materials and Methods

### 2.1. Ethics statement

This study was approved by the Institutional Ethics Committee of Yonsei University College of Medicine, after review of the protocol and procedures employed (4-2013-0261), with all samples collected after obtaining informed consent prior to PN. All patients provided written consent to participate in the current study.

### 2.2. Patients

Urine and serum samples were prospectively collected in 176 consecutive patients who underwent PN at a single institution for single, solid, and enhancing renal tumors between June 2013 and May 2014. All patients exhibited a well-enhancing contralateral kidney evidenced by preoperative computed tomography (CT). PN was performed as previously described using an open or laparoscopic approach according to the surgeons’ preference [14,15]. Ischemia type (none, warm, or cold) and the use of intravenous mannitol were decided according to surgeon discretion and depending on each intraoperative situation. For each patient, the clinical features were prospectively recorded, including sex; age; body mass index (BMI); presence of co-morbidities, namely, diabetes mellitus and cardiovascular disease (CVD); preoperative sCr; preoperative aspects and dimensions used for an anatomical (PADUA) score of renal tumors; estimated blood loss (EBL); ischemia type; and ischemia time. CVD was defined as the presence of preoperative coronary artery disease, heart failure, hypertension, high cholesterol levels, hyperlipidemia, and/or high triglyceride levels. AKI was defined as an increase of sCr of more than 50% or by 0.3 mg% from the baseline within 48 h of surgical insult [16].

### 2.3. Sample collection

Urine samples were collected preoperatively via a urethral Foley catheter inserted after the induction of general anesthesia and at 3, 24, and 48 h after renal pedicle clamp removal. In patients operated without renal pedicle clamping, samples were collected 3, 24, and 48 h after the termination of renorrhaphy. Collected urine samples were immediately transported to the clinical laboratory, where they were stored at -80°C until analysis. Samples were later analyzed for NGAL (R&D systems, Abingdon, UK) and Cr (Roche Diagnostics GmbH, Mannheim, Germany) using commercially available enzyme-linked immunosorbent assays. Serum sample analyses were performed as part of routine clinical practice.

### 2.4. Statistical analysis

Baseline characteristics of the patients were compared using descriptive statistical tests between the open and laparoscopic PN groups. Appropriate comparative tests, e.g., Student’s *t*-test and χ^2^-test, were used to compare continuous and categorical variables. Clinical factors including the AKI rate were also compared between groups with preoperative eGFR <60 and ≥60 mL/min/1.73 m^2^ using descriptive statistical tests such as Student’s *t*-test or χ^2^-test.

To evaluate changes in uNGAL levels following PN between groups with differing postoperative renal function, linear mixed models were used to measure uNGAL changes by time periods for the overall group and according to each clinical subgroup, namely, preoperative eGFR <60 and ≥60 mL/min/1.73 m^2^, open and laparoscopic groups, and groups with and without AKI. All analyses were performed together for absolute values and percent changes from baseline of postoperative uNGAL and normalized uNGAL (the ratio of uNGAL to urine creatinine), with adjustment for preoperative values. Normalized uNGAL was used to avoid the potential dilution effect of uNGAL due to hydration status and the use of intravenous mannitol.

Univariate linear mixed models were used to identify clinical factors—namely, age, sex, BMI, preoperative eGFR, preoperative normalized uNGAL, presence of diabetes and CVD, EBL, AKI, PADUA score, differences in surgical type and ischemia type, ischemia time, and use of mannitol—that were associated with changes in uNGAL and normalized uNGAL, with adjustment for preoperative values.

Univariate linear regression analyses were performed to identify clinical predictors, including the above-mentioned clinical factors and postoperative uNGAL changes, associated with eGFR changes at 6 months postoperatively, with adjustment for preoperative eGFR values. All tests were two-sided, with statistical significance set at *p* < 0.05. Statistical analyses were performed using SAS version 9.2 (SAS Institute Inc., Cary, NC, USA).

## Results

### 3.1. Clinical features

Of the 176 patients, we obtained adequate specimens from 146 (82.9%) patients for incorporation in the final analysis. Patients’ characteristics are presented in Table 1. There were no significant differences between the open and laparoscopic groups, except that laparoscopic PN was more frequently performed with warm ischemia as compared to open PN. Overall, the 10 patients with preoperative eGFR <60 mL/min/1.73 m^2^ were older (median age 65 vs. 54 years, *p* < 0.001) and had a higher incidence of CVD (70% vs. 37%, *p* = 0.040) than those with eGFR ≥60 mL/min/1.73 m^2^. However, there were no differences in sex, BMI, the incidence of diabetes, ischemia type, or the incidence of AKI between the two groups (data not presented).

**Table 1:** Patient characteristics of patients treated with partial nephrectomy via open or laparoscopic approach.

	Open	Laparoscopic	*p*
No.	76	70	
Age	54.5 ± 11.9	52.4 ± 12.9	*0*.*314*
Sex			*0*.*276*
Male	48 (63.2%)	38 (54.3%)	
Female	28 (36.8%)	32 (45.7%)	
Body mass index (kg/m^2^)	24.8 ± 3.4	24.3 ± 2.8	*0*.*379*
Diabetes	8 (10.5%)	10 (14.3%)	*0*.*490*
Cardiovascular disease	34 (44.7%)	23 (32.9%)	*0*.*142*
uNGAL (ng/ml)			
Preoperative	8.02 ± 12.3	6.50 ± 10.2	*0*.*419*
Preoperative normalized	7.20 ± 15.3	5.79 ± 7.5	*0*.*490*
Preoperative sCr (mg/dl)	0.86 ± 0.03	0.84 ± 0.02	*0*.*516*
Preoperative eGFR	80.3 ± 12.4	82.9 ± 9.1	*0*.*151*
AKI	20 (26.3%)	14 (20.0%)	*0*.*367*
Ischemia type			*<0*.*001*
Cold	22 (29.0%)	0 (0%)	
Warm	46 (60.5%)	60 (85.7%)	
None	8 (10.5%)	10 (14.3%)	
Ischemia time (min)	20.1 ± 9.9	22.9 ± 13.7	*0*.*157*
Estimated blood loss	211.8 ± 267.1	231.7 ± 198.5	*0*.*613*
Mannitol use	18 (23.7%)	20 (28.6%)	*0*.*501*
PADUA score			*0*.*165*
6–7	37 (48.7%)	26 (37.1%)	
8–9	35 (46.1%)	35 (50.0%)	
≥10	4 (5.2%)	9 (12.9%)	

Data are number (%) and mean ± S.D.

AKI = acute kidney injury

eGFR = estimated glomerular filtration rate

PADUA = preoperative aspects and dimensions used for an anatomical

sCr = serum creatinine

uNGAL = urinary neutrophil gelatinase-associated lipocalin

### 3.2. Changes in uNGAL and normalized uNGAL following PN

**Fig 1:**
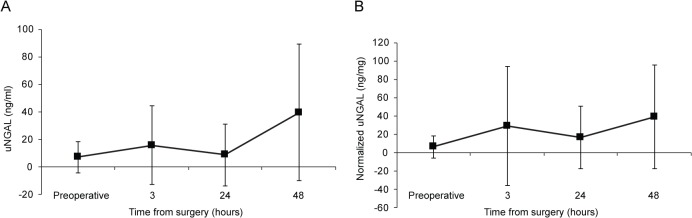
Levels of (A) uNGAL and (B) normalized uNGAL, shown by time periods in the overall group following partial nephrectomy (n = 146).

**Fig 2:**
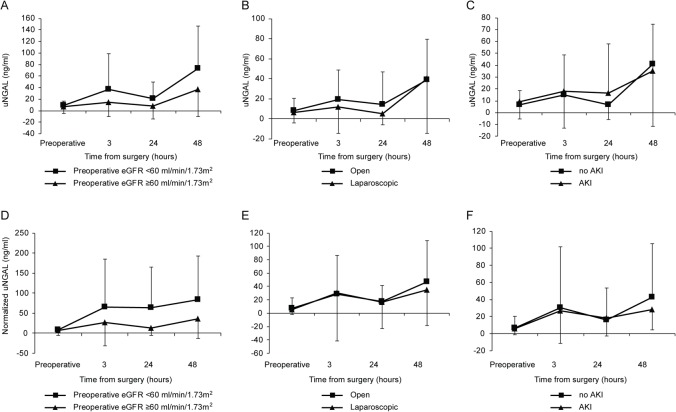
Levels of uNGAL (A, B, and C) and normalized uNGAL (D, E, and F) shown by time periods between subgroups following partial nephrectomy.

As depicted in Fig 1, postoperative uNGAL and normalized uNGAL were increased in the overall group (β = 0.51, 95% CI 0.36–0.66, *p* < 0.001 and β = 0.41, 95% CI 0.18–0.65, *p* < 0.001, respectively). However, as presented in Fig 2, subgroup analyses revealed no significant differences in the changes in uNGAL and normalized uNGAL over time between the subgroups with preoperative eGFR <60 and ≥60 mL/min/1.73 m^2^ (uNGAL: β = -0.42, 95% CI -0.97 –-0.12, *p* = 0.128 and normalized uNGAL: β = -0.54, 95% CI -1.40–0.33, *p* = 0.220), between the open and laparoscopic groups (uNGAL: β = 0.12, 95% CI -0.19–0.42, *p* = 0.451 and normalized uNGAL: β = -0.16, 95% CI -0.65–0.32, *p* = 0.509), and between the groups with AKI and without AKI (uNGAL: β = -0.18, 95% CI -0.53–0.18, *p* = 0.339 and normalized uNGAL: β = -0.30, 95% CI -0.87–0.27, *p* = 0.308). Given the variations in baseline uNGAL and normalized uNGAL levels, we further analyzed these values in respect to percent changes from baseline. However, there were no significant differences in percent changes in uNGAL levels between all subgroups (data not shown).

### 3.3. Predictors of postoperative uNGAL

After adjusting for preoperative uNGAL, univariate linear mixed models demonstrated preoperative normalized uNGAL to be associated with an increase in postoperative uNGAL (β = 0.85, 95% CI 0.78–0.92, *p* < 0.001). No association was observed between the incidences of diabetes, CVD, or AKI; EBL; PADUA score; surgery type; ischemia type; ischemia time; or mannitol use with changes in postoperative uNGAL (Table 2).

**Table 2:** Univariate linear mixed models for predicting postoperative uNGAL, adjusted for preoperative uNGAL.

	NGAL		Normalized uNGAL	
	β (95% CI)*	*p*	β (95% CI)*	*p*
Age	-0.059 (-0.208, 0.09)	*0*.*437*	0.041 (-0.096, 0.178)	*0*.*556*
Sex				
Male	Reference		Reference	
Female	-1.497 (-5.24, 2.246)	*0*.*431*	-1.127 (-4.576, 2.322)	*0*.*519*
Body mass index	-0.123 (-0.713, 0.467)	*0*.*681*	-0.208 (-0.751, 0.335)	*0*.*449*
Preoperative eGFR	-0.009 (-0.177, 0.159)	*0*.*917*	-0.016 (-0.171, 0.139)	*0*.*835*
Preoperative normalized uNGAL	0.849 (0.778, 0.921)	*<0*.*001*	-1.244 (-6.425, 3.937)	*0*.*636*
Diabetes	-3.272 (-8.862, 2.317)	*0*.*249*	0.531 (-2.953, 4.014)	*0*.*764*
Cardiovascular disease	0.186 (-3.596, 3.969)	*0*.*923*	-0.003 (-0.01, 0.004)	*0*.*393*
Estimated blood loss	-0.002 (-0.01, 0.006)	*0*.*602*	0.008 (-4.012, 4.027)	*0*.*997*
AKI	2.39 (-1.958, 6.739)	*0*.*279*	2.387 (-1.151, 5.924)	*0*.*185*
PADUA score				
6–7	Reference		Reference	
8–9	3.091 (-0.75, 6.933)	*0*.*114*	2.387 (-1.151, 5.924)	*0*.*185*
≥10	4.702 (-2.037, 11.44)	*0*.*170*	-2.724 (-8.924, 3.477)	*0*.*387*
Surgery type				
Open	Reference		Reference	
Laparoscopic	-1.127 (-4.816, 2.562)	*0*.*547*	-1.912 (-5.297, 1.474)	*0*.*266*
Ischemia				
None	Reference		Reference	
Cold	-1.636 (-8.746, 5.475)	*0*.*650*	-2.766 (-9.288, 3.757)	*0*.*403*
Warm	-0.879 (-6.582, 4.824)	*0*.*761*	0.04 (-5.193, 5.273)	*0*.*988*
Ischemia				
None	Reference		Reference	
Warm or cold	-1.019 (-6.632, 4.595)	*0*.*720*	-0.44 (-5.612, 4.733)	*0*.*867*
Ischemia time	-0.038 (-0.192, 0.117)	*0*.*633*	-0.061 (-0.203, 0.082)	*0*.*403*
Mannitol use	-2.756 (-6.937, 1.426)	*0*.*195*	-1.833 (-5.694, 2.027)	*0*.*349*

*Estimated change in postoperative uNGAL per unit of predictor

Abbreviations as in Table 1

### 3.4. Predictors of postoperative eGFR at 6 months postoperatively

For 90 patients who were observed until postoperative 6 months, univariate linear regression models were used to evaluate clinical factors indicative of eGFR at postoperative 6 months. Preoperative sCr level was associated with decreased eGFR (β = -16.503, 95% CI -28.457 –-4.549, *p* = 0.007) at 6 months postoperatively. The onset of AKI was also associated with a decrease of 8.73 mL/min/1.73 m^2^ (β = -8.73, 95% CI -12.69 –-4.77, *p* < 0.001) in eGFR at 6 months postoperatively. The uNGAL level at 3 h after renal pedicle clamp removal was associated with an increased eGFR (β = 0.07, 95% CI 0.01–0.13, *p* = 0.023) at 6 months postoperatively. However, no significant association existed between normalized uNGAL level at 3 h and eGFR at 6 months postoperatively (β = 0.038, 95% CI -0.002–0.077, *p* = 0.060; Table 3).

**Table 3:** Univariate linear regression models for predicting eGFR at postoperative 6 months.

	β (95% CI)*	*p*
Age	-0.098 (-0.262, 0.066)	*0*.*237*
Sex		
Male	Reference	
Female	2.112 (-1.607, 5.831)	*0*.*262*
Body mass index	-0.254 (-0.914, 0.406)	*0*.*447*
Diabetes	1.440 (-5.016, 7.895)	*0*.*659*
Cardiovascular disease	-2.785 (-6.903, 1.332)	*0*.*182*
Estimated blood loss	-0.002 (-0.009, 0.006)	*0*.*693*
Preoperative sCr	-16.503 (-28.457, -4.549)	*0*.*007*
AKI	-8.731 (-12.69, -4.766)	*<0*.*001*
PADUA score		
6–7	Reference	
8–9	-0.064 (-3.818, 3.689)	*0*.*973*
≥10	-5.411 (-12.37, 1.548)	*0*.*126*
Surgery type		
Open	Reference	
Laparoscopic	-0.249 (-3.957, 3.459)	*0*.*894*
Ischemia		
None	Reference	
Cold	-3.171 (-10.37, 4.030)	*0*.*384*
Warm	-1.063 (-7.574, 5.448)	*0*.*746*
Ischemia		
None	Reference	
Warm or cold	-1.630 (-8.020, 4.761)	*0*.*614*
Ischemia time	-0.071 (-0.231, 0.090)	*0*.*388*
Mannitol use	-1.862 (-5.895, 2.170)	*0*.*361*
uNGAL postoperative 3hr	0.072 (0.010, 0.133)	*0*.*023*
uNGAL postoperative 24hr	0.018 (-0.102, 0.137)	*0*.*770*
uNGAL postoperative 48hr	0.019 (-0.038, 0.076)	*0*.*505*
Normalized uNGAL postoperative 3hr	0.038 (-0.002, 0.077)	*0*.*060*
Normalized uNGAL postoperative 24hr	0.066 (-0.016, 0.147)	*0*.*110*
Normalized uNGAL postoperative 48hr	0.045 (-0.002, 0.092)	*0*.*060*

*Estimated change in postoperative eGFR per unit of predictor

Abbreviations as in Table 1.

## Discussion

To date, there have been no objective clinical predictors for quantifying the degree of AKI and long-term renal function; until recently, NGAL has been reported as a useful marker for the early identification of ischemic and/or tubular damage [10,17–22]. However, not many human studies have documented the efficacy of NGAL in quantifying AKI following PN in patients with normal contralateral kidney. Two studies that have incorporated patients who underwent open PN have reported conflicting results. Initial results reported by Abassi et al. demonstrated uNGAL as a quantitative marker for AKI based on the results of 27 patients who underwent open PN [11]. In contrast, Sprenkle et al. showed negative results for the usefulness of uNGAL since they observed that the levels of uNGAL after open PN were comparable to those after thoracic surgery [12]. Unfortunately, both studies are limited by a relatively small number of patients and the inclusion of only open PN, wherein most surgeries were performed with the use of maximal renal protective techniques. Therefore, these observations may be due to minimal renal damage rather than the inability of uNGAL to determine the degree of renal injury. In this study that comprised a relatively large cohort of open and laparoscopic PN cases, we addressed whether the level of uNGAL altered significantly after PN and whether it could be utilized as a quantitative marker for AKI after PN.

We postulated that if the change in NGAL level is a useful marker for quantifying AKI after PN, there would be a difference between subgroups. Accordingly, we analyzed changes in uNGAL levels according to time periods following PN between various subgroups, namely, patients with preoperative eGFR <60 and ≥60 mL/min/1.73 m^2^, open and laparoscopic PN, and patients with and without AKI. We also reviewed whether clinical factors and uNGAL changes were associated with eGFR changes at 6 months postoperatively, assuming that if uNGAL were a useful marker for AKI after PN, it would eventually reflect long-term renal function.

In the present study, only 6.8% of patients had preoperative eGFR <60 mL/min/1.73 m^2^; therefore, the uNGAL changes over time following PN in our overall patients were considered likely to represent postoperative uNGAL changes in the unilateral renal injury model in patients with normal contralateral kidney and relatively good preoperative renal function. We observed increased uNGAL levels over time following PN in the entire patient cohort; however, the uNGAL changes over time did not differ among the subgroups, those who may have different postoperative renal function. Notably, there were no differences in the postoperative uNGAL changes between patients who underwent open and laparoscopic PNs, probably due to comparable clinical confounders between the two groups, except for the type of ischemia. Our findings did not agree with previous findings as we failed to demonstrate any differences in the uNGAL changes over time between groups with preoperative eGFR <60 and ≥60 mL/min/1.73 m^2^ (40% vs. 22%, *p* = 0.240) [12]. Moreover, uNGAL changes over time did not show any differences even between the groups with and without AKI.

The negative results for uNGAL as a marker of AKI between the clinical subgroups were in accordance with previous results, which failed to identify any clinical factors associated with the levels of uNGAL [12]. In our study, only preoperative normalized uNGAL was associated with an increase in postoperative uNGAL level (β = 0.85). Moreover, preoperative sCr level and the presence of AKI were both associated with decreases in the postoperative 6-month eGFR, rather than a change in uNGAL itself. Unexpectedly, the level of uNGAL at 3 h following renal pedicle clamp removal was associated with the level of eGFR at 6 months postoperatively. Although this finding was counterintuitive, its clinical usefulness seems to be limited as evidenced by the low β value of 0.07, and the observation that normalized uNGAL level at 3 h was not associated with an increased eGFR at 6 months postoperatively. The linear regression analysis for predicting eGFR at postoperative 6 months was performed for only 90 patients who were followed until postoperative 6 months, and the postoperative follow-up period itself seemed to be relatively short to assess long-term renal function. Indeed, studies with a larger cohort of patients with at least 1 year follow-up period is warranted to clearly evaluate clinical factors indicative of long-term renal function.

Based on these results, we suggest that an increase in uNGAL may not reflect the degree of AKI severity or postoperative renal function in patients with a normal contralateral kidney and a relatively good preoperative renal function. Moreover, unlike previous studies, our results may be applied to not only PN but also to ureteroscopic or shockwave lithotripsy procedures and in other unilateral renal injury models in healthy patients [23]. Parekh et al. also reported that the changes of several urinary biomarkers for AKI, including uNGAL, were relatively small and were not associated with the extent of structural alteration during ischemia, duration of ischemia, or the transient increases of creatinine in patients undergoing PN. They suggested that the lesser discriminative power of the biomarkers in patients with PN may be caused by the lack of more severe and sustained injury by surgical procedures, and that urinary biomarkers may not be useful for predicting early functional AKI in this setting [13].

The current study has certain limitations. First, our results are in accordance with those of Sprenkle et al., wherein uNGAL did not appear to be a useful marker for detecting renal injury in healthy patients treated by PN [12]. However, we did not observe any differences between groups with preoperative eGFR <60 and ≥60 mL/min/1.73 m^2^ in terms of the incidence of AKI and changes in uNGAL levels at different time points. This discrepancy may arise from the limited number of patients (6.8%) with preoperative eGFR <60 mL/min/1.73 m^2^ in our study. Further studies with a larger cohort of patients with poor preoperative renal function are in need to evaluate the clinical usefulness of uNGAL. Second, we collected all urine samples through a urethral Foley catheter for convenience and timed-collection of preoperative and postoperative urine samples. However, the change of intravascular volume or blood pressure levels following the induction of general anesthesia may have influenced the baseline levels of uNGAL. For these reasons, a comprehensive analysis was performed for both postoperative uNGAL and normalized uNGAL in an effort to overcome such potential bias. Moreover, urine samples were collected through a Foley catheter, and therefore, uNGAL levels may have been underestimated due to the urine volume excreted from the normal contralateral kidney. The uNGAL level in the urine sample collected through a ureteral catheter from the ipsilateral kidney undergoing partial nephrectomy may hold greater clinical relevance for AKI severity. However, a useful clinical marker should be easily applicable in routine clinical practice; therefore, we evaluated the usefulness of uNGAL levels in urine samples obtained from the urethral catheter. Third, the postoperative follow-up period of 6 months was relatively short for assessing long-term renal function. Moreover, we were unable to incorporate the relative volume of preserved, vascularized renal tissue into our predictive models, while this factor has been suggested to predict postoperative renal function [24]. Lastly, our study included a prospectively collected, larger cohort of PN cases as compared to previous studies. Nevertheless, variations in baseline patient features, namely, eGFR (preoperative eGFR <60 and ≥60 mL/min/1.73 m^2^), comorbidity, age, tumor complexity, surgical factors (open versus laparoscopic, use of mannitol, ischemia time and type, and surgeon experience), and the limited number of patients in each subgroup, is a clear limitation. For a better understanding of the clinical usefulness of uNGAL, further studies with a stricter inclusion criterion, preferably with a larger number of patients will be needed.

### Conclusions

While uNGAL levels may increase after PN, the changes in uNGAL levels did not differ among the subgroups that may exhibit different postoperative renal function. Therefore, we suggest that the usefulness of uNGAL for quantifying AKI severity and predicting postoperative renal function is limited in patients with normal contralateral kidney and relatively good preoperative renal function.
